# Long-term Outcomes Among Patients With Advanced Kidney Disease Who Forgo Maintenance Dialysis: A Systematic Review

**DOI:** 10.1001/jamanetworkopen.2022.2255

**Published:** 2022-03-14

**Authors:** Susan P. Y. Wong, Tamara Rubenzik, Leila Zelnick, Sara N. Davison, Diana Louden, Taryn Oestreich, Ann L. Jennerich

**Affiliations:** 1 Health Services Research and Development Center, Veterans Affairs Puget Sound Health Care System, Seattle, Washington; 2 Division of Nephrology, University of Washington, Seattle; 3 Divisions of Nephrology and Geriatrics, Gerontology and Palliative Care, University of California, San Diego; 4 Division of Nephrology, University of Alberta, Edmonton, Canada; 5 Health Sciences Library, University of Washington, Seattle; 6 Division of Pulmonary, Critical Care and Sleep Medicine, University of Washington, Seattle

## Abstract

**Importance:**

An understanding of the long-term outcomes of patients with advanced chronic kidney disease not treated with maintenance dialysis is needed to improve shared decision-making and care practices for this population.

**Objective:**

To evaluate survival, use of health care resources, changes in quality of life, and end-of-life care of patients with advanced kidney disease who forgo dialysis.

**Evidence Review:**

MEDLINE, Embase (Excerpta Medica Database), and CINAHL (Cumulative Index of Nursing and Allied Health Literature) were searched from inception through December 3, 2021, for all English language longitudinal studies of adults in whom there was an explicit decision not to pursue maintenance dialysis. Two investigators independently reviewed all studies and selected those reporting survival, use of health care resources, changes in quality of life, or end-of-life care during follow-up. Studies of patients who initiated and then discontinued maintenance dialysis and patients in whom it was not clear that there was an explicit decision to forgo dialysis were excluded. One author abstracted all study data, of which 12% was independently adjudicated by a second author (<1% error rate).

**Findings:**

Forty-one cohort studies comprising 5102 patients (range, 11-812 patients) were included in this systematic review (5%-99% men; mean age range, 60-87 years). Substantial heterogeneity in study designs and measures used to report outcomes limited comparability across studies. Median survival of cohorts ranged from 1 to 41 months as measured from a baseline mean estimated glomerular filtration rate ranging from 7 to 19 mL/min/1.73 m^2^. Patients generally experienced 1 to 2 hospital admissions, 6 to 16 in-hospital days, 7 to 8 clinic visits, and 2 emergency department visits per person-year. During an observation period of 8 to 24 months, mental well-being improved, and physical well-being and overall quality of life were largely stable until late in the illness course. Among patients who died during follow-up, 20% to 76% had enrolled in hospice, 27% to 68% died in a hospital setting and 12% to 71% died at home; 57% to 76% were hospitalized, and 4% to 47% received an invasive procedure during the final month of life.

**Conclusions and Relevance:**

Many patients who do not pursue dialysis survived several years and experienced sustained quality of life until late in the illness course. Nonetheless, use of acute care services was common and intensity of end-of-life care highly variable across cohorts. These findings suggest that consistent approaches to the study of conservative kidney management are needed to enhance the generalizability of findings and develop models of care that optimize outcomes among conservatively managed patients.

## Key Points

**Question:**

What are the long-term outcomes of patients with advanced kidney disease who do not pursue maintenance dialysis?

**Findings:**

In this systematic review of 41 cohort studies comprising 5102 adults with advanced kidney disease who did not pursue dialysis, limited available evidence suggests that many patients survived several years and experienced sustained quality of life until late in their illness course. However, use of acute care services was common, and there was substantial disparity in access to supportive care near the end of life across cohorts.

**Meaning:**

These findings suggest that advances in research and health care delivery are needed to optimize outcomes among patients who are not treated with dialysis.

## Teaser

This systematic review assesses cohort studies reporting survival, use of health care resources, quality of life, and end-of-life care among patients with advanced chronic kidney disease who forgo maintenance dialysis.

## Introduction

Conservative kidney management is a planned, holistic, and person-centered approach to care for patients with stages 4 to 5 advanced chronic kidney disease (CKD) who do not wish to pursue maintenance dialysis.^1^ It includes “interventions to delay progression of kidney disease and minimize risk of adverse events or complications; shared decision making; active symptom management; detailed communication including advance care planning; psychological support; social and family support; [and] cultural and spiritual domains of care.”^2^^(p7)^ Desire for a more conservative approach to treating patients with advanced CKD has galvanized efforts around the world to develop the evidence base to support the care of these patients.

Toward this end, several systematic reviews and meta-analyses^3,4,5,6,7^ have been conducted comparing outcomes between patients treated with dialysis and those treated conservatively. They show that dialysis is associated with longer survival compared with conservative approaches^3,4,5,6^ but that these survival advantages are attenuated with increasing age and comorbidity.^7^ Patients treated conservatively also spend less time in the hospital and die there less often compared with patients receiving dialysis,^6^ and early changes in quality of life appear similar between treatment groups.^7,8^

Although the findings of these prior studies help to inform shared decision-making about treatment of advanced CKD, they are restricted to studies comparing groups treated with dialysis and those treated conservatively. As a result, prior systematic reviews and meta-analyses reflect only a small fraction of the patients who forgo dialysis described in the literature and provide only a limited view of the clinical course of patients to guide ongoing management and anticipatory guidance to patients who have already decided that they will not pursue dialysis. To support a deeper understanding of the long-term outcomes of patients with advanced CKD who do not pursue dialysis, we performed a systematic review of longitudinal studies reporting survival, use of health care resources, quality of life, and end-of-life care of patients with advanced CKD who did not pursue dialysis.

## Methods

### Data Sources

This systematic review was performed in accordance with the Preferred Reporting Items for Systematic Reviews and Meta-Analyses (PRISMA) guideline and is registered in PROSPERO (CRD42020156086). An experienced medical librarian (D. L.) performed a comprehensive search of MEDLINE, Embase (Excerpta Medica Database), and CINAHL (Cumulative Index of Nursing and Allied Health Literature) for all English language publications pertaining to patients with advanced CKD who did not pursue maintenance dialysis from inception through January 27, 2020, with a search update through December 3, 2021. We used database-specific subject heading terms and a range of text words (*conservative*, *non-dialysis*, *palliative*, *supportive*, and *medical management*) previously used in the literature to describe this approach to care^9^ (eMethods in the Supplement) to locate all potentially relevant articles.

### Study Selection

We included all longitudinal studies that enrolled patients 18 years or older with advanced CKD in whom an explicit decision was made not to pursue maintenance dialysis. We selected studies reporting survival, use of health care resources (ie, all-cause hospitalization and in-hospital days, emergency department visits, and clinic visits), changes in quality of life, or end-of-life care (ie, hospice enrollment, place of death, and hospitalization and invasive procedures during the final month of life) during follow-up and a baseline measure of estimated glomerular filtration rate (eGFR) from which outcomes were measured. We excluded studies of patients who initiated and then discontinued maintenance dialysis, those that did not include information on baseline eGFR, and those of patients in whom it was unclear that an explicit decision to forgo dialysis was made. Case reports, qualitative studies, and the gray literature were excluded.

The results of search queries were imported into Covidence (Veritas Health Innovation Ltd) for screening and study selection. Two authors (S.P.Y.W. and T.R.) independently screened titles and abstracts and reviewed full-text articles to determine study eligibility. Disagreements were resolved through consensus by another author (L.Z. or A.L.J.).

### Data Extraction

Data extraction was performed by 1 reviewer (S.P.Y.W.) using a standardized data extraction form. For studies comparing multiple treatment groups, we collected information only on groups of patients in whom there was a decision not to pursue dialysis from each study. From each study, we collected information on study design, year of publication, country of origin, sample size, study inclusion criteria, clinical setting, and whether patients were cared for in a dedicated care pathway for those not planning to be treated with dialysis or in usual nephrology care settings. Because nearly all the studies did not report information on the race and ethnicity of its study participants, this information was not collected. We recorded baseline age, sex, eGFR, and distribution of comorbidities of study participants. We recorded measures of survival, use of health care resources, and changes in quality of life. We also collected information on patterns of end-of-life care for study participants who died during study follow-up. We contacted study authors by email to obtain any missing data. One investigator (T.O.) independently reviewed the full text of 5 randomly selected studies (12%) to confirm accuracy of data extraction (<1% errors found).

### Data Synthesis and Analysis

Owing to the heterogeneity in study designs, patient populations, approaches to care, and measures used across studies, we opted not to meta-analyze collected data; we herein provide a narrative synthesis of reported outcomes. For our primary outcome, we evaluated the median (IQR) survival of patients and the baseline mean eGFR from which survival was measured. For studies reporting only a threshold eGFR value (eg, <20 mL/min/1.73 m^2^), the closest value (ie, 19 mL/min/1.73 m^2^) was used in place of mean values. Median eGFR values were used in place of mean values when the latter were not reported. For studies that did not report median and/or IQR measures of survival, these values were abstracted from reported Kaplan-Meier survival curves, estimated using reported mortality rates assuming an exponential distribution, and/or calculated using reported means of survival and their SDs.^10^ Studies that had insufficient information to estimate median survival or were limited to only patients who died during follow-up were not included in survival analyses. Preplanned subgroup assessment of survival by study region (Asia, Australia, continental Europe, North America, and the UK), year of study publication (before 2010, 2010-2015, and after 2015), mean age of the study cohort (70-79 and ≥80 years), and approach to care (as part of general nephrology care vs a dedicated care pathway) were also performed.

As secondary outcomes, we assessed use of health care resources, trajectories of quality of life, and end-of-life care of patients. Evaluation of these secondary measures by study region, publication date, age of cohort, and approach to care could not be performed owing to the low number of studies in each category.

## Results

### Study Characteristics

**Figure 1.**
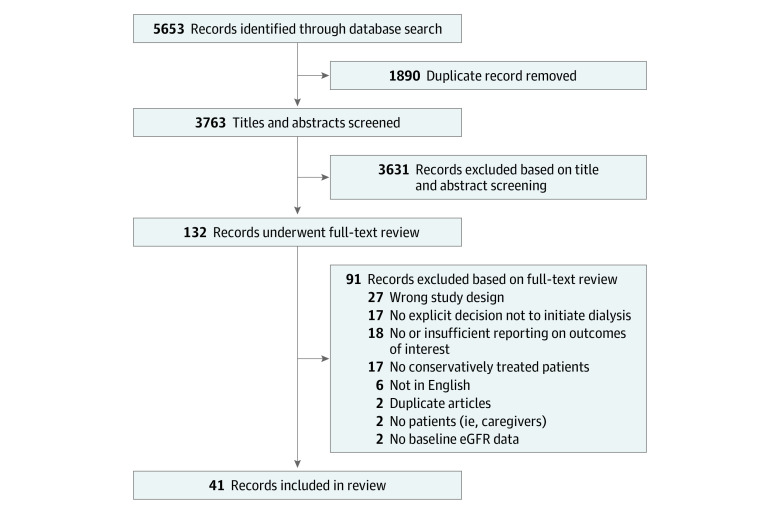
PRISMA Flow Diagram of Study Selection eGFR indicates estimated glomerular filtration rate.

The literature search yielded 5653 references, of which the full text was reviewed in 132 (Figure 1). A total of 41 cohort studies comprising 5102 patients (study size range, 11-812 patients; 5%-99% men; mean age range, 60-87 years) were included in this review (eTable 1 in the Supplement).^11,12,13,14,15,16,17,18,19,20,21,22,23,24,25,26,27,28,29,30,31,32,33,34,35,36,37,38,39,40,41,42,43,44,45,46,47,48,49,50,51^ No clinical trials were identified in our search.

### Survival

**Figure 2.**
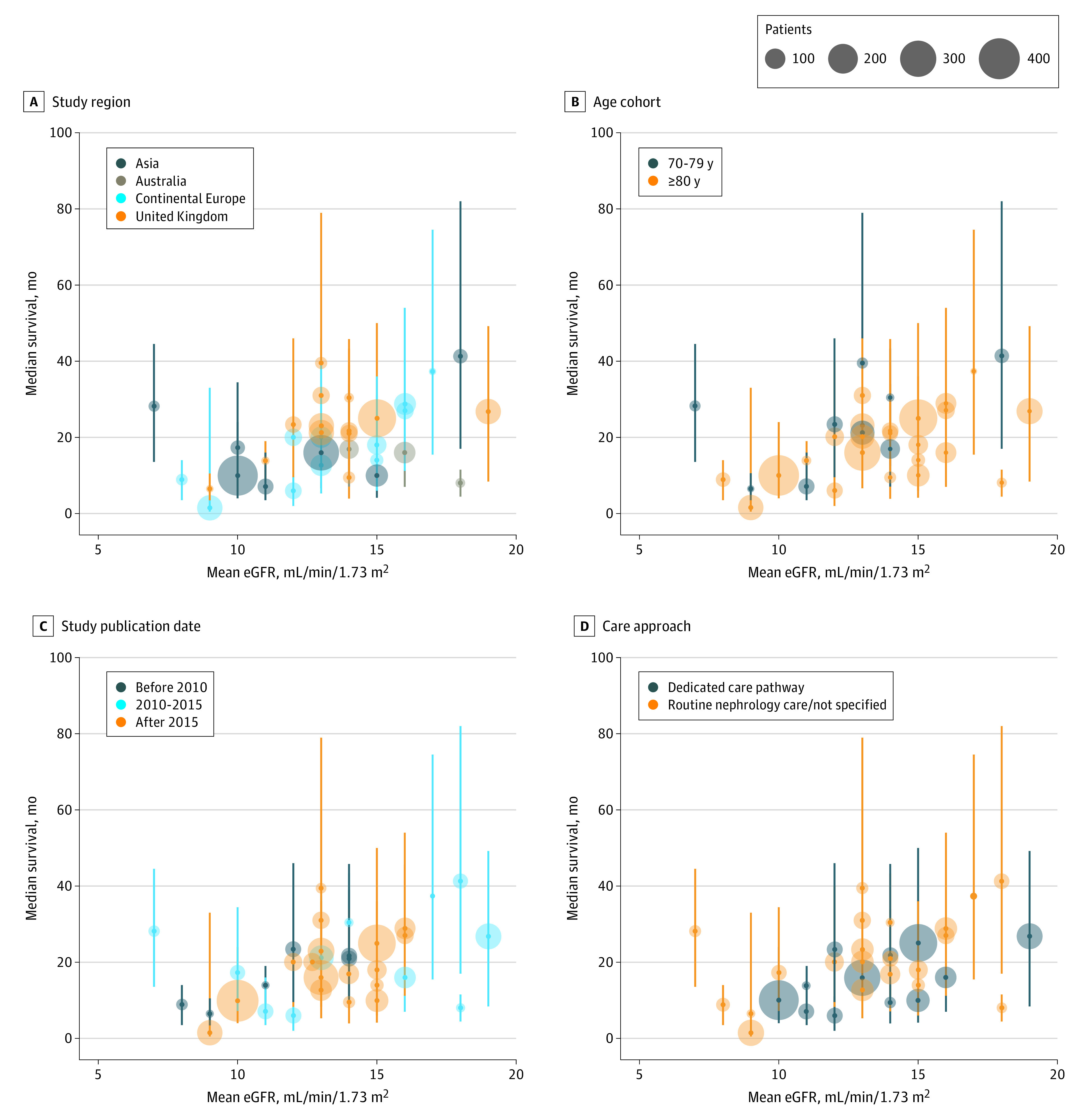
Median Survival of Cohorts With Conservative Management According to Baseline Estimated Glomerular Filtration Rate (eGFR) Median survival varied widely by study region (A), age group (B), year of study publication (C), and approach to conservative care (D). Vertical lines represent IQRs.

Thirty-four studies (3754 patients)^11,12,13,14,15,16,17,18,20,21,22,23,25,26,27,29,30,31,34,35,36,37,38,39,40,41,42,43,44,45,46,47,48,50^ provided information on median survival and IQR or sufficient information to estimate these values (eTable 2 in the Supplement). The range of median survival of cohorts was 1 to 41 months as measured from a baseline mean eGFR range of 7 to 19 mL/min/1.73 m^2^ (Figure 2).

The median survival of cohorts ranged from 1 to 31 months in the UK (13 studies; 1320 patients),^12,15,16,18,20,22,25,31,34,35,36,43,50^ 6 to 37 months in continental Europe (11 studies; 1021 patients),^21,23,29,37,38,39,40,45,46,47,48^ 7 to 41 months in Asia (7 studies; 1147 patients),^13,14,26,27,41,42,44^ and 8 to 17 months in Australia (3 studies; 258 patients).^11,17,30^ The wide ranges in median survival for cohort members in these regions corresponded to the wide ranges in baseline mean eGFR (7-18 mL/min/1.73 m^2^ for Asia, 14-18 mL/min/1.73 m^2^ for Australia, 8-17 mL/min/1.73 m^2^ for continental Europe, and 9-19 mL/min/1.73 m^2^ for the UK). No studies published in North America provided sufficient information to estimate median survival of cohort members in this region. Median survival was 6 to 22 months and baseline mean eGFR was 8 to 14 mL/min/1.73 m^2^ for cohorts published before 2010 (6 studies; 317 patients),^12,20,23,31,43,50^ 6 to 37 months and 7 to 19 mL/min/1.73 m^2^ for cohorts published from 2010 to 2015 (11 studies; 851 patients),^11,13,15,17,18,22,39,41,42,44,45^ and 1 to 39 months and 9 to 16 mL/min/1.73 m^2^ for cohorts published after 2015 (17 studies; 2586 patients).^14,16,21,25,26,27,29,30,34,35,36,37,38,40,46,47,48^ Younger cohorts aged 70 to 79 years (9 studies; 607 patients)^13,15,18,30,34,42,43,44,50^ had a median survival of 7 to 41 months, and cohorts 80 years or older (25 studies; 3186 patients)^11,12,14,16,17,19,20,21,23,25,26,27,29,30,31,35,36,37,38,39,40,45,46,47,48^ had a median survival of 1 to 37 months despite overlapping ranges of baseline mean eGFR (7-18 and 8-18 mL/min/1.73 m^2^, respectively). Patients who were treated in usual nephrology care settings or who had received an unspecified approach to care (22 studies; 1886 patients)^15,16,17,18,20,21,23,29,30,34,36,37,38,39,40,41,42,43,44,46,47,48^ had a median survival of 1 to 39 months and baseline mean eGFR of 7 to 17 mL/min/1.73 m^2^, whereas patients managed in a dedicated care pathway (12 studies; 1944 patients)^11,12,13,14,22,25,26,27,31,35,45,50^ had a median survival of 1 to 27 months and mean baseline eGFR of 10 to 19 mL/min/1.73 m^2^.

### Quality of Life

Eight studies (500 patients)^11,18,25,33,34,40,41,46^ measured changes in quality of life among patients (Table 1) using different standardized instruments (36-Item Short Form Health Survey [SF-36], Kidney Disease Quality of Life Short Form [KDQOL-SF], and EuroQol-5D Scale). Studies also ascertained ancillary measures pertaining to quality of life, including symptom burden (Memorial Symptom Assessment Scale and Palliative Care Outcome Scale–Symptoms), functional status (Barthel Index), frailty (Timed Up-and-Go), mental health symptoms (Hospital Anxiety and Depression Scale), life satisfaction (Satisfaction With Life Scale), and self-rated overall health (visual analog scale). The period of observation for changes in quality of life ranged from 8 to 24 months across studies.

**Table 1.** Quality-of-Life Trajectories

Source	No. of patients	Follow-up, mo^a^	Change in status				
			Overall QOL	Physical well-being	Mental well-being	Symptom burden	Self-rated health
Brown et al,^11^ 2015	122^b^	10^c^	NR	SF-36 physical health worse	SF-36 mental health better	MSAS worse, POS-S worse	NR
Da Silva-Gane et al,^18^ 2012	30	15	NR	SF-36 physical health no change	SF-36 mental health better	SWLS no change, HADS depression no change, HADS anxiety no change	NR
Kilshaw et al,^25^ 2016	41	11^c^	EQ5D worse	NR	NR	TUG worse, Barthel Index no change	NR
Murtagh et al,^33^ 2011	49	8	NR	NR	NR	MSAS worse, POS-S worse	NR
Phair et al,^34^ 2018	42	12	EQ5D worse	NR	NR	NR	NR
Rubio Rubio et al,^40^ 2019	64	NR	NR	SF-36 physical health no change	SF-36 mental health better	NR	NR
Seow et al,^41^ 2013	63	24	NR	KDQOL-SF physical health worse	KDQOL-SF mental health better	NR	NR
van Loon et al,^46^ 2019	89	NR	EQ5D worse	NR	NR	NR	VAS worse

Abbreviations: EQ5D, EuroQol-5D Scale; HADS, Hospital Anxiety and Depression Scale; KDQOL-SF, Kidney Disease Quality of Life Short Form; MSAS, Memorial Symptom Assessment Scale; NR, not reported; POS-S, Palliative Care Outcome Scale–Symptoms; QOL, quality of life; SF-36, 36-Item Short Form Health Survey; SWLS, Satisfaction With Life Scale; TUG, Timed Up-and-Go; VAS, visual analog scale.

^a^ Unless otherwise indicated, data are expressed as mean.

^b^ Response rates for each survey ranged from 55% to 74%.

^c^ Indicates median values.

Studies measuring quality of life using the EuroQol-5D Scale reported small decrements (3 studies; 172 patients)^25,34,46^ in overall quality of life during an 11- to 12-month time frame. Although 1 study using the SF-36 found that most of its cohort members (12 of 19 patients [63%]) reported worse physical well-being during a 12-month follow-up period,^11^ 3 other studies (157 patients) using either the SF-36 or the KDQOL-SF reported stable physical well-being during a 15- to 24-month follow-up period^18,40^ and then decline thereafter.^41^ All studies (4 studies; 279 patients)^11,18,40,41^ using either the SF-36 or the KDQOL-SF reported improvements in mental well-being among their cohort members.

Although in 1 study most patients reported improvement in symptom burden during a 12-month follow-up period on the Memorial Symptom Assessment Scale (12 of 21 [57%]) and Palliative Care Outcome Scale–Symptoms (49 of 69 [71%]) instruments,^11^ in another study examining symptom burden in the final year of life limited to decedents (49 patients),^33^ Memorial Symptom Assessment Scale and Palliative Care Outcome Scale–Symptoms scores were generally stable until the final 3 months of life, after which symptom burden increased before death. One study (30 patients)^18^ reported stable symptoms of anxiety and depression (Hospital Anxiety and Depression Scale) and unchanged sense of life satisfaction (Satisfaction With Life Scale) among its cohort members during a 15-month follow-up. One study (41 patients)^25^ reported sustained ability to perform activities of daily living (Barthel Index) but increasing risk of falls (Timed Up-and-Go) among its cohort members during an 11-month follow-up. One study (89 patients)^46^ reported lower self-rated health (visual analog scale) over time among its cohort members.

### Use of Health Care Resources

Ten studies (570 patients)^12,19,21,34,36,39,42,45,46,48^ provided information on use of health care resources during follow-up (Table 2). Whereas 1 study (42 patients)^42^ reported a mean of 4 hospital admissions per person-year and 38 in-hospital days per person-year among its cohort members, the other studies (528 patients)^12,19,21,34,36,39,45,46,48^ reported approximately 1 to 2 hospital admissions per person-year and 6 to 16 in-hospital days per person-year. It was also reported that patients experienced approximately 7 to 8 clinic visits per person-year (2 studies; 207 patients)^36,48^ and 2 emergency department visits per person-year (1 study; 76 patients).^45^

**Table 2.** Use of Health Care Resources

Source	No. of patients	Follow-up, mo^a^	No. of resources used per person-year^a^			
			Hospitalizations	In-hospital days	Emergency department visits	Clinic visits
Carson et al,^12^ 2009	29	14	NR	16	NR	NR
De Biase et al,^19^ 2008	11	15	2^b^	11^b^	NR	NR
García Testal et al,^21^ 2021	54	NR	NR	15	NR	NR
Phair et al,^34^ 2018	42	12	NR	11	NR	NR
Raman et al,^36^ 2018	81	NR	NR	10	NR	8^a^
Rodriguez Villarreal et al,^39^ 2014	20	19^b^	1	NR	NR	NR
Shum et al,^42^ 2014	42	23^b^	4	38	NR	NR
Teruel et al,^45^ 2015	76	8	1	NR	2	NR
van Loon et al,^46^ 2019	89	6	2^b^	8^b^	NR	NR
Verberne et al,^48^ 2018	126	NR	1	6	NR	7

Abbreviation: NR, not reported.

^a^ Unless otherwise indicated, data are expressed as mean.

^b^ Indicates median value.

### End-of-Life Care

Fourteen studies (1709 patients)^12,19,22,24,26,28,30,40,42,43,45,49,50,51^ provided information on end-of-life care for cohort members who died during follow-up (Table 3). Reported rates of hospice enrollment (20%-76%),^22,24,28,51^ hospitalization during the final month of life (57%-76%),^22,51^ in-hospital death (27%-68%),^12,19,22,24,26,30,40,43,45,49,51^ and in-home death (12%-71%),^19,30,40,45,50^ were wide ranging. One study (39 patients)^42^ that defined intensive procedures during the final month of life as receipt of operative or endoscopic procedures, mechanical ventilation, cardiopulmonary resuscitation, and/or artificial enteral nutrition reported 47% of decedents received such procedures. Another study (812 patients)^51^ that examined only rates of mechanical ventilation, cardiopulmonary resuscitation, and artificial enteral nutrition found that only 4% of decedents received these intensive procedures during the final month of life.

**Table 3.** End-of-Life Care

Source	No. of decedents	Decedents, %					
		Hospitalization during final month of life	Intensive procedures during final month of life	Hospice enrollment	In-hospital death	In-home death	Other place of death
Carson et al,^12^ 2009	25	NR	NR	NR	36	NR	Home or hospice, 40
De Biase et al,^19^ 2008	5	NR	NR	NR	40	60	NR
Hussain et al,^22^ 2013	77	76	NR	76	47	NR	Hospice,18; nursing home, 12
Kamar et al,^24^ 2017	103	NR	NR	25	27	NR	Home or LTC, 32; other, 17
Kwok et al,^26^ 2016	226	NR	NR	NR	47	NR	Emergency department, 4
Lovell et al,^28^ 2017	146	NR	NR	20	46	34	NR
Morton et al,^30^ 2016	72	NR	NR	NR	42	12	Hospice, 14; nursing home, 6
Rubio Rubio et al,^40^ 2019	38	NR	NR	NR	68	32	NR
Shum et al,^42^ 2014	39	NR	47	NR	NR	NR	NR
Smith et al,^43^ 2003	34	NR	NR	NR	35	NR	Home or hospice, 65
Teruel et al,^45^ 2015	48	NR	NR	NR	31	50	Inpatient palliative care, 19
Verberne et al,^49^ 2020	56	NR	NR	NR	32	NR	NR
Wong et al,^50^ 2007	28	NR	NR	NR	NR	71	NR
Wong et al,^51^ 2018	812	57	4	39	41	NR	NR

Abbreviations: LTC, long-term care; NR, not reported.

## Discussion

This systematic review provides a comprehensive summary of the long-term outcomes of patients with advanced CKD who did not pursue maintenance dialysis. Our findings offer insights into directions for research and clinical care to improve survival, quality of life, use of health care resources, and end-of-life care for members of this population (eFigure in the Supplement).

Although research on conservative kidney management has been growing, available evidence is best regarded as very limited and preliminary. We observed a high degree of heterogeneity in study designs and measures used to assess outcomes across studies, thus limiting comparability across studies and the generalizability of our findings. In contrast, there have been national and international efforts to establish unified approaches to epidemiological surveillance of kidney replacement therapy and its outcomes.^52,53^ An important step toward advancing research on conservative kidney management is to implement similar approaches to the study and reporting of its practices and outcomes.

Our findings challenge the common misconception that the only alternative to dialysis for many patients with advanced CKD is no care^54^ or death.^55^ Despite the advanced ages and significant comorbid burden of cohorts in this study, most patients survived several years after the decision to forgo dialysis was made. We also found that mental well-being improved over time and that physical well-being and overall quality of life were largely stable until late in the illness course. These findings not only suggest that conservative kidney management may be a viable and positive therapeutic alternative to dialysis, they also highlight the strengths of its multidisciplinary approach to care and aggressive symptom management. Still, there was substantial variation in survival across cohorts according to regions, time periods, and approaches to care. We suspect that the observed variation is likely attributable to differences in local health systems and practices and their changes over time.^56,57,58,59^ Hence, our findings also underscore the need to develop models of care that optimize outcomes for members of this population who have the potential to live well and for quite some time without dialysis.

Although patients who forgo dialysis spend less time in the hospital than patients undergoing maintenance dialysis,^13,36,43,47^ we found that many still frequently used acute care services. Previous studies^52,60^ have shown that many existing health systems are not optimally configured to support the needs of patients who do not wish to pursue dialysis and that patients and their clinicians often fall back on acute care services when there are gaps in community-based and preventive care services.^56^ Collectively, our findings point to an area of focus in efforts to develop the care infrastructure that can help patients avoid or mitigate the impact of a health crisis.

We observed striking disparities in access to quality end-of-life care across cohorts. Although among some cohorts, most patients received hospice care, avoided burdensome procedures near the end-of-life, and died at home, for other cohorts, only a minority accessed supportive care near the end of life. One of the distinct benefits afforded by conservative kidney management is that patients who choose this option experience less intensive care near the end of life than patients who receive dialysis.^6,22,30,42,43,51^ However, it also is recognized that patients who forgo dialysis receive hospice services less often and die in the hospital setting more often than other patients in the same health system but with other serious illness, such as terminal cancer.^51^ More research is needed to understand and overcome the barriers to supportive care for this population.

### Limitations

The present study should be interpreted within the context of the following limitations. First, in studies for which mean eGFR, median survival, and IQR were estimated based on available data reported, these values are imprecise and limit the reliability of our findings. Second, the present review includes only patients in whom there had been an explicit decision made to not undergo dialysis and for whom conservative management was likely a planned approach to care. Thus, our findings do not provide insights on the experiences of patients with untreated advanced CKD, those who are unsure about dialysis, or those who had not yet faced decisions about dialysis in their illness course.^61,62,63^ The present study also does not reflect the experiences of patients in developing countries or other resource-poor settings where conservative options may not be widely available.^59^ Third, although we eliminated duplicate studies from this review, owing to similar studies conducted at the same site during overlapping time periods, there were several studies that may have included duplicate cases.^32,33,47,48^ These cases likely constituted a very small proportion (<4%) of the total number of patients included in this review and would unlikely have a substantial effect on our main findings. Fourth, lack of consistency is apparent in the terminology used to describe caring for patients not treated with dialysis in published literature.^64^ Although we used multiple different terms in our literature search to identify all relevant articles pertaining to this approach to care, articles that used alternate terms might have been missed. Fifth, approaches to quality and bias assessments of cohort studies recommended for systematic reviews pertain to studies comparing 2 or more treatment groups^65^ and therefore were not appropriate for the present analyses that focused on the long-term outcomes of only patients who had chosen not to pursue dialysis. To facilitate reader appraisal of studies included in this review, we provide extensive descriptions of cohort design, characteristics, and measures collected for each study to present the relevance, consistency, comprehensiveness, and depth of detail provided on the populations and outcomes studied.^66^

## Conclusions

Despite substantial heterogeneity across studies on the long-term outcomes of patients with advanced CKD who forgo maintenance dialysis, this systematic review found that patients could survive several years and experienced improvements in their mental well-being in addition to sustaining physical well-being and overall quality of life until late in their illness course. Nonetheless, use of acute care services was common and intensity of end-of-life care was highly variable across cohorts of patients. Collectively, our findings demonstrate the need to implement systematic and unified research methods for conservative kidney management and to develop models of care and the care infrastructure to advance practice and outcomes of conservative kidney management.

## Notes

Supplement. **eMethods.** Search Strategy for Medline (EBSCO) **eTable 1.** Description of Conservatively Managed Cohorts **eTable 2.** Median Survival and Mean eGFR of Conservatively Managed Cohorts **eFigure.** Summary of Findings of Current Systematic Review and Recommendations to Advance Conservative Kidney Management
