# Supplementary material for: Long-term Outcomes Among Patients With Advanced Kidney Disease Who Forgo Maintenance Dialysis: A Systematic Review
Source: JAMA Netw Open. 2022 Mar 14;5(3):e222255. doi: 10.1001/jamanetworkopen.2022.2255 (PMC9907345; doi:10.1001/jamanetworkopen.2022.2255)
Supplement: Supplement. — eMethods. Search Strategy for Medline (EBSCO) eTable 1. Description of Conservatively Managed Cohorts eTable 2. Median Survival and Mean eGFR of Conservatively Managed Cohorts eFigure. Summary of Findings of Current Systematic Review and Recommendations to Advance Conservative Kidney Management [file jamanetwopen-e222255-s001.pdf]

Supplementary Online Content

Wong SPY, Rubenzik T, Zelnick L, et al. Long-term outcomes among patients with advanced kidney disease who forgo maintenance dialysis: a systematic review. *JAMA Netw Open*. 2022;5(3):e222255. doi:10.1001/jamanetworkopen.2022.2255

eMethods. Search Strategy for Medline (EBSCO)
eTable 1. Description of Conservatively Managed Cohorts
eTable 2. Median Survival and Mean eGFR of Conservatively Managed Cohorts
eFigure. Summary of Findings of Current Systematic Review and Recommendations to Advance Conservative Kidney Management

This supplementary material has been provided by the authors to give readers additional information about their work.

© 2022 Wong SPY et al. *JAMA Network Open*.

eMethods 1. Search Strategy for Medline (EBSCO)

(((MM “Kidney Failure, Chronic+” OR MM "Renal Insufficiency, Chronic+" OR TI (“Chronic Kidney Failure” OR “Chronic Renal Failure” OR “Chronic Renal Insufficiency” OR “advanced chronic kidney disease” OR “advanced chronic kidney diseases” OR “chronic kidney disease” OR “chronic kidney diseases” OR CKD OR “advanced kidney disease” OR “kidney failure” OR “End Stage Kidney Disease” OR “End Stage Renal Disease” OR “End Stage Renal Failure” OR “Late Stage Kidney Disease” OR “Late Stage Renal Disease” OR “Late Stage Renal Failure” OR ESRD OR ESKD))

AND

(MM “Terminal Care+” OR MM “Hospice Care+” OR MM “Palliative Care+” OR MM “Conservative Treatment+” OR MM “Withholding Treatment+” OR MM “Treatment Refusal+” OR TI (“Terminal Care” OR “End of Life Care” OR “Life Care End” OR “Life Care Ends” OR “Hospice Care” OR “Hospice Program” OR “Hospice Programs” OR “Bereavement Care” OR “palliative care” OR “palliative treatment” OR “palliative treatments” OR “palliative therapy” OR “supportive care” OR “conservative treatment” OR “conservative treatments” OR “conservative management” OR “conservative managements” OR “conservative therapy” OR “conservative therapies” OR “conservative care” OR “conservatively managed” OR “conservatively treated”) OR AB (“Terminal Care” OR “End of Life Care” OR “Life Care End” OR “Life Care Ends” OR “Hospice Care” OR “Hospice Program” OR “Hospice Programs” OR “Bereavement Care” OR “palliative care” OR “palliative treatment” OR “palliative treatments” OR “palliative therapy” OR “supportive care” OR “conservative treatment” OR “conservative treatments” OR “conservative management” OR “conservative managements” OR “conservative therapy” OR “conservative therapies” OR “conservative care” OR “conservatively managed” OR “conservatively treated”) OR TI conservative OR TI conservatively OR (TI ((forgo OR forgoing OR without OR withhold* OR withheld OR decline* OR declining OR refuse OR refusing OR refusal* OR refused) N3 (dialysis OR hemodialysis))) OR (AB ((forgo OR forgoing OR without OR withhold* OR withheld OR decline* OR declining OR refuse OR refusing OR refusal* OR refused) N3 (dialysis OR hemodialysis))) OR TI (“maximal medical management” OR “withholding treatment” OR “withholding treatments” OR “withholding therapy” OR “treatment refusal” OR “refusing treatment”) OR AB (“maximal medical management” OR “withholding treatment” OR “withholding treatments” OR “withholding therapy” OR “treatment refusal” OR “refusing treatment”) OR ((MM “Renal Dialysis+” OR TI “renal dialysis” OR TI “kidney dialysis” OR TI “hemodialysis”) AND TI (forgo OR forgoing OR without OR withhold* OR decline OR declining OR refuse OR refusing OR conservative OR conservatively))))

OR

(AB (“Chronic Kidney Failure” OR “Chronic Renal Failure” OR “Chronic Renal Insufficiency” OR “advanced chronic kidney disease” OR “advanced chronic kidney diseases” OR “chronic kidney disease” OR “chronic kidney diseases” OR CKD OR “advanced kidney disease” OR “kidney

© 2022 Wong SPY et al. *JAMA Network Open*.

failure” OR “End Stage Kidney Disease” OR “End Stage Renal Disease” OR “End Stage Renal Failure” OR “Late Stage Kidney Disease” OR “Late Stage Renal Disease” OR “Late Stage Renal Failure” OR ESRD OR ESKD) AND (TI (“conservative management” OR “conservative treatment” OR “conservative care” OR “conservative therapy” OR “conservatively managed” OR “conservatively treated”) OR AB (“conservative management” OR “conservative treatment” OR “conservative care” OR “conservative therapy” OR “conservatively managed” OR “conservatively treated”))))

NOT

(MH "Case Reports" OR PT "case reports" OR “case report” OR “case reports” OR “case study” OR “case studies”)

© 2022 Wong SPY et al. *JAMA Network Open*.

eTable 1. Description of Conservatively Managed Cohorts

**STable 1. Description of conservatively managed cohorts**						
**Author, year**	**Country, setting (sites)**	**Study design; aims**	**Inclusion criteria**	**Dedicated pathway? Services provided**	**N (mean age, male %)**	**Comorbidities**
Brown, 2015	Australia, Renal Supportive Care Clinic (single)	PRO; compare survival and quality of life between older patients who plan and plan not to receive dialysis	NR	Yes. Provided separate from and in addition to usual nephrology care; staffed by palliative care specialist and senior renal palliative care nurse with support of dietician and social worker as needed; focused on symptom management, advance care planning, discussions on prognosis.	122 (82y, 55%)	CHF 36%, Dem 11.5%, DM 53%
Carson, 2009	UK, Low Clearance Clinic (single)	PRO; compare survival, hospitalization rates and location of death for patients who chose either maximal conservative management or renal replacement therapy	age ≥70, eGFR <30, reached eGFR >10.8 by end of follow-up	Yes. MDT including nephrologist, clinical nurse specialist, dietician, and social worker; includes home visit by social worker; facilitated access to hospice and home palliative care; optimization of Hgb, HTN, volume status, and cholesterol levels; K balance through diet; Ca and P balance targeted to pruritis	29 (81y, 59%)	DM 14%
Chan, 2010	HK, Renal Palliative Care Clinic (single)	RETRO; describe reasons for decision, management, symptom burden, cause of death,	eGFR <15 with DM or eGFR <10, no AKI, underwent	Yes. Provided by dedicated nephrologists; control of uremic symptoms; flexible follow-up appointments; blood transfusions as needed	74 (72y, 35%)	metastatic CA 1%, CHF 28%, COPD 8%, CVA 35%, Dem 1%, DM

© 2022 Wong SPY et al. *JAMA Network Open*.

		survival, place of death, and code status of incident patients who opted for supportive management	dialysis assessment ;			68%, IHD 24%, Liv 1%, PVD 8%
Chan, 2020	HK, Renal Palliative Care Clinic (single)	RETRO; evaluate the impact of a comprehensive program of psychosocial interventions for fluid management among patients who opt for conservative management	age > 60, eGFR <15, enrolled in clinic's comprehensive fluid management program	Yes. Provided jointly by nephrologist and palliative care physician; psychoeducation program provided by palliative care nurse and dietician and focused on diet self-efficacy with the aim to improve patient's knowledge and adherence to self-care behaviors and continuous monitoring of their symptoms.	138 (82y, 51%)	CA 20%, CHF 47%, CVA 26%, DM 63%, HTN 84%, IHD 57%, Lung 11%,
Chandna, 2011	UK, renal clinics (multi)	RETRO; compare survival of conservatively managed patients to those of a contemporaneous group of patients on hemodialysis	age ≥18y, first renal clinic visit with eGFR >15, had at least 1 eGFR 10-15 and all subsequent eGFR <15, no AKI	No. MDT in liaison with community, primary care and hospice services as appropriate; full medical treatment except dialysis	155 (78y 59%)	DM 36%

© 2022 Wong SPY et al. *JAMA Network Open*.

Chandna, 2016	UK, renal clinics (multi)	RETRO; examine the extent to which the rate of kidney functional decline influenced choice of dialysis or conservative kidney management and how eGFR affects the survival of patients in both these treatment groups.	age ≥75y, eGFR <15, first nephrology clinic visit with eGFR >10, no AKI	No. MDT in liaison with community, primary care and hospice services as appropriate; full medical treatment except dialysis	158 (82y, 60%)	DM 30%
Cheikh Hassan, 2015	Australia, Renal Supportive Care Clinic (single)	RETRO; compare the safety and effectiveness of gabapentin for pruritus or restless leg syndrome in conservatively managed patients and patients on hemodialysis	age ≥18y, eGFR<90, at least 2 nephrology clinic visits, has CKD-related pruritis and/or restless leg syndrome treated with gabapentin	NR	34 (80y, 56%)	CVD 27%, DM 59%
Da Silva-Gane, 2012	UK, Low Clearance Clinics (multi)	PRO; compare quality of life trajectories and survival in patients opting for conservative kidney	eGFR <30, has decision-making capacity,	No. MDT of nephrologists, specialist nurses, renal counselors, social workers, dietitians, and community and	30 (78y, 70%)	NR

© 2022 Wong SPY et al. *JAMA Network Open*.

		management and those opting for dialysis.	adequate English proficiency	hospice services; full medical treatment except dialysis		
De Baise, 2008	Italy, renal clinic (single)	RETRO; assess compliance with the Italian clinical practice guidelines for renal failure, comorbidities, hospital stays, and psychometric and quality of life indicators of the patients and their caregivers who chose conservative therapy over dialysis.	age ≥75y, GFR <15, eligible for dialysis, no prior dialysis, clinical follow-up >6months	No. Care in accordance with Italian Society of Nephrology guidelines; dietary counseling on K, Na and fluid intake; diet supplemented with keto-analogues as needed; lab tests and clinic visits every 4–6 weeks.	11 (82y, 64%)	NR
Ellam, 2009	UK, NR (NR)	RETRO; investigate factors influencing survival in a cohort of conservatively managed patients	eGFR <15, no AKI	NR	69 (80y*, 58%)	CA 9%, CHF 28%, COPD 20%, CVD 19%, DM 38%, Dem 10%, IHD 32%, PVD 1%
Garcia Testal, 2020	Spain, nephrology department (single)	RETRO; compare the survival of patients on renal replacement therapy with those on conservative treatment and	age >80y, received nephrology consultatio n	No. Full medical treatment except dialysis	54 (87y, 30%)	CA 28%. DM 43%, HTN 93%, IHD 31%

© 2022 Wong SPY et al. *JAMA Network Open*.

		healthcare resources required by both groups				
Hussain, 2013	UK, Conservative Management Program (single)	RETRO; compare survival, hospital admissions and palliative care access between patients who chose renal replacement therapy or conservative management.	age >70y, eGFR <20, eligible for dialysis, no AKI	Yes. MDT including palliative medicine consultant; management of fluid and electrolyte balance, Hgb, renal bone disease; care emphasis on quality of life, symptom control and advance care planning.	172 (82y, 51%)	CA 19%, CVD 16%, Dem 7%, DM 40%, IHD 36%, Liv 2%, PVD 17%
Joly, 2003	France, renal unit (single)	RETRO; identify factors influencing therapeutic recommendations between groups proposed either dialysis or conservative treatment	age ≥80y, eGFR <10, no prior dialysis, no AKI	NR. Close cooperation with the family doctor; fluid management including ultrafiltration without dialysis as needed; pharmacologic and nonpharmacologic measures for uremic symptoms, pain; attention to psychologic, social, and spiritual concerns.	37 (84y, 38%)	CA 14%, CHF 51%, CVD 22%, DM 22%, IHD 49%, PVD 22%

© 2022 Wong SPY et al. *JAMA Network Open*.

Kamar, 2017	Canada, Conservative Care Program (single)	RETRO; describe the characteristics of patients treated with conservative care	age ≥75y, eGFR <30, prior nephrology clinic visit, died during follow-up	Yes. Provided by palliative medicine-trained nephrologist, advanced care planning nurse clinician, dedicated conservative care nurses, social worker and dietician as needed; education on prognosis; care guided by patient- and family-specific goals and wishes and symptoms; clinic, telephone and home visits; bereavement services for families.	154 (81y, 46%)	metastatic CA 5%, CHF 26%, COPD 21%, CVD 25%, DM 56%, IHD 25%, Liv 1% PVD 18%
Kilshaw, 2016	UK, Conservative Management Clinic (single)	PRO; describe the trajectory of functional status within our conservative management population	eGFR <30, has decision-making capacity	Yes. MDT by nephrologist, conservative management specialist nurse, social worker, anemia nurse and dietician and physiotherapist; palliative care service referrals as needed.	41 (83y, 44%)	NR
Kwok, 2016a	HK, Renal Palliative Care Progam (single)	RETRO; evaluate the symptoms and interventions initiated in the last 2 weeks of life, health care service utilization, and causes of death of patients in a Renal Palliative Care Program.	NR	Yes. MDT care; structured advance care planning; supported by a palliative home care team in the community; admitted to designated beds under the palliative care team for management of acute needs and end-of-life care as needed	335 (77y, NR)	CA 9%, CHF 30%, COPD 5%, CVD 29%, Dem 12%, DM 66%, IHD 30%, Liv 11%, PVD 6%
Kwok, 2016b	UK, renal team (single)	RETRO; identify factors associated	Evaluated for	Yes. MDT by physicians, specialist nurses, social workers, dietitians,	432 (80y, 42%)	CA 11%, CHF 29%, CVD

© 2022 Wong SPY et al. *JAMA Network Open*.

		with survival in patients managed with either renal replacement therapy or conservative treatment	creatinine level >350 mmol/L with diabetes mellitus or >400 mmol/L without diabetes; age ≥65y, eGFR <15, clinic follow-up throughout study period	occupational therapists, and physiotherapists; medical and sociopsychological management; telephone hotline for symptom control.		29%, Dem 13%, DM 60%, IHD 31%, Pulm 4%, Liv 5%, PVD 4%
Lovell, 2017	UK, Conservative Management Service (single)	RETRO; understand patterns of place of death in patients with end-stage kidney disease	age ≥75y, eGFR <30, prior nephrology clinic visit, died during follow-up	Yes. Care in accordance with International Society of Nephrology guidelines; interventions to delay progression of disease, shared decision making, active symptom management, advance care planning, psychological support, social and family support, and cultural and spiritual domains of care.	146 (NR, NR)	NR

© 2022 Wong SPY et al. *JAMA Network Open*.

Moranne, 2018	France, renal centers (multi)	PRO; determine uptake of dialysis, dialysis start conditions, and survival among patients who decided for or against dialysis or postponed this decision.	age >75y, eGFR <20, at least 1 nephrology clinic visit, no prior dialysis or kidney transplant, no AKI	NR	124 (NR, NR)	DM 40%, CA 14%, CHF 42%, CVD 15%
Morton, 2016	Australia, renal units (multi)	PRO; determine the proportion of patients who switched to dialysis after confirmed plans for conservative care and compare survival and end-of-life care among patients choosing conservative care with those initiating renal replacement therapy	eGFR <15, no AKI, no prior kidney transplant or dialysis	NR	102 (79y, 51%)	NR
Murtagh, 2007	UK, renal units (multi)	RETRO; compare survival and hospitalization of non-dialysis pathway patients with end-stage kidney disease managed through a Renal Supportive Care	age >75y	Yes. MDT of physicians, specialist nurses, counselors, and dieticians; additional time and resources for education, psychosocial, medical decision-making support	77 (83y*, 66%)	NR

© 2022 Wong SPY et al. *JAMA Network Open*.

		Group to patients who received standard nephrologist-led conservative care				
Murtagh, 2011a	UK, renal units (multi)	PRO; determine the functional trajectory in the last year of life in end-stage renal disease managed conservatively without dialysis	eGFR <15, had decision-making capacity	NR	74 (81y, 51%)	NR
Murtagh, 2011b	UK, renal units (multi)	PRO; describe trajectory of symptoms and patient health-related concerns over the last year of life for patients with managed conservatively without dialysis	eGFR <15, has decision-making capacity, died during follow-up	Yes. Dedicated renal palliative care nurse specialists with nephrology staff support as needed; clinic, telephone and home-based care; cooperation with primary care and specialist palliative care.	49 (81y, 49%)	CA 18%, CHF 33%, Coll 4%, DM 37%, IHD 35%, PVD 6%, other 33%
Phair, 2018	UK, renal units (multi)	PRO; describe cost and quality of life in patients receiving conservative management for end-stage kidney disease	eGFR ≤15	NR	42 (78y, 40%)	NR
Pyart, 2020	UK, renal unit (single)	RETRO; explore how patient characteristics influenced whether patients chose to	eGFR <20; attended pre-dialysis education;	Yes. A holistic approach involving MDT to help maintain patient quality of life, coordinated care with community physicians,	375 (*83y, 56%)	NR

© 2022 Wong SPY et al. *JAMA Network Open*.

		receive renal replacement therapy or opted for maximal conservative management and survival from the time of their final choice		geriatricians, and palliative care providers		
Raman, 2018	UK, Nephrology Secondary Care Clinic or nephrology inpatient ward in a health system (single)	PRO; evaluated whether dialysis has survival advantage compared to conservative care in people who were medically suitable for dialysis therapy.	age >75y, eGFR <15, no prior CHF, no prior cardiac arrest, no prior CA, no Karnofsky Performance Score <60, no dementia, no AKI, no plan for kidney transplant	NR	81 (84y, 56%)	CHF 46%, COPD 14%, CVD 19%, DM 30%, IHD 25%, PVD 15%
Ramspek, 2021	Netherland, teaching hospital (single)	RETRO; develop a prediction tool that predicts the mortality risk for the same patient for both dialysis and	age ≥65y, eGFR <30, prior nephrology care, no AKI	NR	126 (*82y, 54%)	CA 14%, CHF 27%, DM 45%, IHD 44%, PVD 51%

© 2022 Wong SPY et al. *JAMA Network Open*.

		conservative care from the time of treatment decision				
Reindl-Schwaighofer, 2017	Austria, tertiary care hospital (single)	RETRO; compare survival of conservatively managed patients to patients who started dialysis	age >65y, eGFR <10, no AKI, no CA	NR	174 (81y, 5%)	CA 16%, COPD 18%, DM 34%, IHD 70%, Liv 13%, PVD 51%
Rodriguez Villarreal, 2014	Spain, pre-dialysis clinic (single)	PRO; identify factors influencing decision-making between treatment with dialysis or conservative care	age >75y, eGFR <30	No. Full medical treatment except dialysis	20 (83y, NR)	Card 50%, DM 30%, PVD 40%
Rubio Rubio, 2019	Spain, hospital nephrology department (single)	PRO; analyze variables predictive of mortality in patients opting for conservative kidney management and changes in health-related quality of life in this population	age >75y	NR	82 (85y, 56%)	NR
Seow, 2013	Singapore, renal clinics and ward of a tertiary hospital (single)	PRO; compared health-related quality of life of patients who remained on conservative management versus	age ≥75 or age-adjusted CCI ≥8, eGFR 8-12	NR	63 (60y, 56%)	NR

© 2022 Wong SPY et al. *JAMA Network Open*.

		those who switched to renal replacement therapy				
Shum, 2014	HK, tertiary care hospital (single)	RETRO; compare survival of peritoneal dialysis versus conservative management in adults	age ≥65y, eGFR <15, assessed for dialysis	No. Management of fluid, Hgb and electrolyte balance and uremic symptoms; telephone hotline for symptom control.	42 (75y, 43%)	CA 5%, CHF 17%, CVD 29%, Dem 2%, DM 67%, IHD 29%, Liv 5%, Lung 2%
Smith, 2003	UK, renal unit (single)	PRO; study factors influencing the recommendation for palliative non-dialytic treatment and subsequent outcome	no prior dialysis, evaluated for chronic renal failure	No. MDT of nephrologists, specialist nurses, renal counselors, social workers, dietitians, and community and hospice services; full medical treatment except dialysis	34 (71y, 38%)	DM 43%
Szeto, 2011	HK, nephrology clinic (single)	PRO; study the life expectancy of patients treated conservatively	eligible for dialysis	NR	63 (71y, 51%)	Card 24%; CVA 15.8%, DM 65%, PVD 8%
Teruel, 2015	Spain, Home Care Program (single)	PRO; describe implementation of a home-based renal palliative care program	eGFR <30, not eligible for dialysis, referred to hospital palliative care team	Yes. Home-based team directed by primary care provider and in conjunction with nephrology department; care coordination by palliative care unit; monthly visits and lab tests every 3 months.	76 (81y, 61%)	NR

© 2022 Wong SPY et al. *JAMA Network Open*.

van Loon, 2019	Netherlands, hospitals (multi)	PRO; assess trajectory of quality of life in patients starting dialysis or maximal conservative management	age ≥65y, eGFR <15, decision for conservative care within prior 3 months, has decision-making capacity, good Dutch proficiency, no other terminal condition	NR	89 (82y, 56%)	NR
Verberne, 2016	Netherlands, teaching hospital (single)	RETRO; compare survival of patients choosing either conservative management or renal replacement therapy	age ≥70y, no AKI, received nephrology care	No. MDT care; full medical treatment except dialysis	107 (83y, 55%)	Card 77%
Verberne, 2018	Netherlands, teaching hospital (single)	RETRO; compare healthcare costs between patients who have chosen conservative care versus dialysis	age ≥70y, eGFR <30, received nephrology care	No. MDT care; full medical treatment except dialysis	126 (83y, 54%)	NR

© 2022 Wong SPY et al. *JAMA Network Open*.

Verberne, 2020	Netherlands, teaching hospital (single)	RETRO; compare outpatient and inpatient hospital utilization and costs overall and per hospital department in patients on a conservative care or dialysis pathway	age ≥70y, eGFR <30, received nephrology care, clinic follow-up during study period	No. MDT care; full medical treatment	100 (83y, 56%)	CA 12%, CHF 29%, DM 45%, IHD 45%, PVD 51%
Wong, 2007	UK, renal unit (single)	PRO; evaluate hospitalization rates, survival, prognostic factors influencing the survival of non-dialytic treatment patients and if the Renal Association Standards for renal anemia, renal biochemical parameters, and blood pressure are reached in practice in this population	Prior nephrology care	Yes. Care in accordance with International Society of Nephrology guidelines; interventions to delay progression of disease, shared decision making, active symptom management, advance care planning, psychological support, social and family support, and cul	73 (79y*, 51%)	NR
Wong, 2018	US, national health system (multi)	RETRO; compare end-of-life care between patients who receive dialysis, those in whom there was a decision to not pursue dialysis and those who were	2 outpatient eGFR <15 >90 days apart, died during follow-up	NR	812 (75y, 99%)	CA 32%, CHF 42%, COPD 29%, CVD 13%, Dem 9%, DM 53%, IHD 46%, PAD 17%, Liv 2%

© 2022 Wong SPY et al. *JAMA Network Open*.

	discussing/preparing for dialysis prior to death	

Abbreviations: AKI, acute kidney injury; CA, cancer; Ca, calcium; Card, unspecified cardiovascular disease; CCI, Charlson Comorbidity Index; Coll, collagen vascular disease; CHF, congestive heart failure; COPD, chronic obstructive pulmonary disease; CVD, cerebrovascular disease; Dem, dementia; DM, diabetes mellitus; eGFR, estimated glomerular filtration rate (ml/min/1.73m2); EOL, end-of-life; Hgb, hemoglobin; HK, Hong Kong; HTN, hypertension; IHD, ischemic heart disease; K, potassium; Liv, unspecified liver disease; Lung, unspecified lung disease; MDT, multidisciplinary team; NR, not reported; PVD, peripheral vascular disease; P, phosphate; PRO, prospective cohort; QOL, quality of life; RETRO retrospective cohort; UK, United Kingdom; US, United States.
* median age

Abbreviations: AKI, acute kidney injury; CA, cancer; Ca, calcium; Card, unspecified cardiovascular disease; CCI, Charlson Comorbidity Index; Coll, collagen vascular disease; CHF, congestive heart failure; COPD, chronic obstructive pulmonary disease; CVD, cerebrovascular disease; Dem, dementia; DM, diabetes mellitus; eGFR, estimated glomerular filtration rate (ml/min/1.73m2); EOL, end-of-life; Hgb, hemoglobin; HK, Hong Kong; HTN, hypertension; IHD, ischemic heart disease; K, potassium; Liv, unspecified liver disease; Lung, unspecified lung disease; MDT, multidisciplinary team; NR, not reported; PVD, peripheral vascular disease; P, phosphate; PRO, prospective cohort; QOL, quality of life; RETRO retrospective cohort; UK, United Kingdom; US, United States.
* median age

© 2022 Wong SPY et al. *JAMA Network Open*.

eTable 2. Median Survival and Mean eGFR of Conservatively Managed Cohorts

Study, year	n	eGFR (ml/min/1.73m2)			Survival (months)				
		mean	SD		mean	SD	median	IQR	95% CI
Brown, 2015	122	16	9		16	NR	^16	7-39	NR
Carson, 2009	29	&11	NR		NR	NR	14	$9-19	NR
Chan, 2010	74	11	7		NR	NR	7	$4-16	NR
Chan, 2020	138	≤15	NR		NR	NR	10	*4-20	NR
Chandna, 2011	155	13	1		NR	NR	21	$13-38	NR
Chandna, 2016	158	13	1		NR	NR	23	$12-36	20-27
Cheikh Hassan, 2015	34	18	11		NR	NR	8	4-12	NR
Da Silva-Gane, 2012	30	14	3		NR	NR	30	$13-43	NR
Ellam, 2009	69	<15	NR		NR	NR	21	$11-39	NR
Garcia Testal, 2020	54	≤15	NR		19	16	14	6-31	NR
Hussain, 2013	172	<20	NR		NR	NR	27	$8-49	NR
Joly, 2003	43	8	1		NR	NR	9	$4-14	4-10
Kilshaw, 2016	41	14	4		NR	NR	*1	*0-2	NR
Kwok, 2016a	335	13	4		NR	NR	16	*4-24	NR
Kwok, 2016b	432	10	3		NR	NR	10	$4-24	8-12
Moranne, 2018	124	&13	NR		NR	NR	*13	*5-25	NR
Morton, 2016	102	<15	NR		NR	NR	*17	*7-34	NR
Murtagh, 2007	77	<15	NR		NR	NR	22	$9-46	NR
Phair, 2018	42	13	NR		NR	NR	*39	*16-79	NR
Pyart, 2020	375	&15	NR		NR	NR	25	*10-50	21-28
Raman, 2018$	81	13	2		NR	NR	$31	$17-46	NR
Ramspek, 2021	126	13	5		NR	NR	*20	*8-40	NR
Reindl-Schwaighofer, 2017	174	<10	NR		NR	NR	1	$1-33	0-11
Rodriguez Villarreal, 2014	20	17	6		NR	NR	*37	*15-75	NR
Rubio Rubio, 2019	82	16	3		NR	NR	$27	$11-54	20-34
Seow, 2013	63	&10	NR		NR	NR	*17	*7-34	NR
Shum, 2014	42	7	2		NR	NR	28	14	45
Smith, 2003	26	9	2		NR	NR	6	$4-11	NR
Szeto, 2011	63	18	6		NR	NR	41	NR	33-49
Teruel, 2015$	76	12	6		NR	NR	$6	$2-11	NR
Van Loon, 2019	89	12	4		NR	NR	*20	*8-40	NR
Verberne, 2016	107	15	5		NR	NR	18	8	36
Verberne, 2018	126	16	5		NR	NR	29	17-46	NR
Wong, 2007	73	12	NR		NR	NR	23	*10-46	NR

© 2022 Wong SPY et al. *JAMA Network Open*.

* indicates that median survival and/or IQR estimated using proportion surviving at time (t) assuming exponential distribution
$ indicates that median survival and/or IQR abstracted from Kaplan-Meier curves
^ indicate that mean values used in place of median values of survival
& median values of eGFR
< threshold values of eGFR
Abbreviations: eGFR, estimated glomerular filtration rate; NR, not reported

© 2022 Wong SPY et al. *JAMA Network Open*.

eFigure. Summary of Findings of Current Systematic Review and Recommendations to Advance Conservative Kidney Management

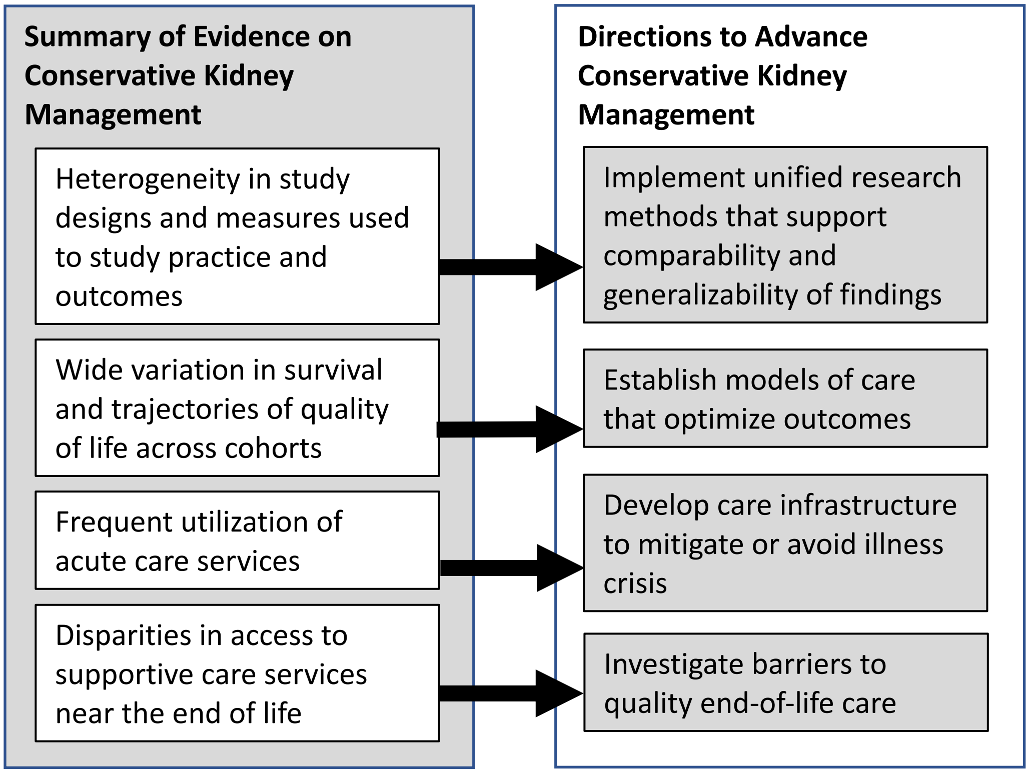

© 2022 Wong SPY et al. *JAMA Network Open*.
